# Usefulness of position emission tomography/computed tomography in a case of sarcoidosis with multiorgan involvement

**DOI:** 10.1002/ccr3.5358

**Published:** 2022-02-13

**Authors:** Norihiko Amano, Soshi Takahashi, Saori Hatachi, Shunichi Kumagai

**Affiliations:** ^1^ 36833 The Center for Rheumatic Disease Shinko Hospital Kobe Japan

**Keywords:** bone sarcoidosis, FDG PET/CT, massive splenomegaly, multiorgan, sarcoidosis

## Abstract

Sarcoidosis, a systemic inflammatory disease of unknown etiology, can affect any site in the body. A bone lesion was unexpectedly detected by fluorodeoxyglucose position emission tomography/computed tomography (FDG PET/CT) in a patient with multiorgan sarcoidosis. FDG PET/CT should be considered for the detection of clinically silent lesions of sarcoidosis.

## Graphical abstract

Sarcoidosis can affect any site in the body, and the clinical course is extremely variable and often asymptomatic. FDG PET/CT is useful in the diagnosis and workup of sarcoidosis, especially for bone lesions.

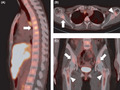

## 1 CASE PRESENTATION

**FIGURE 1:**
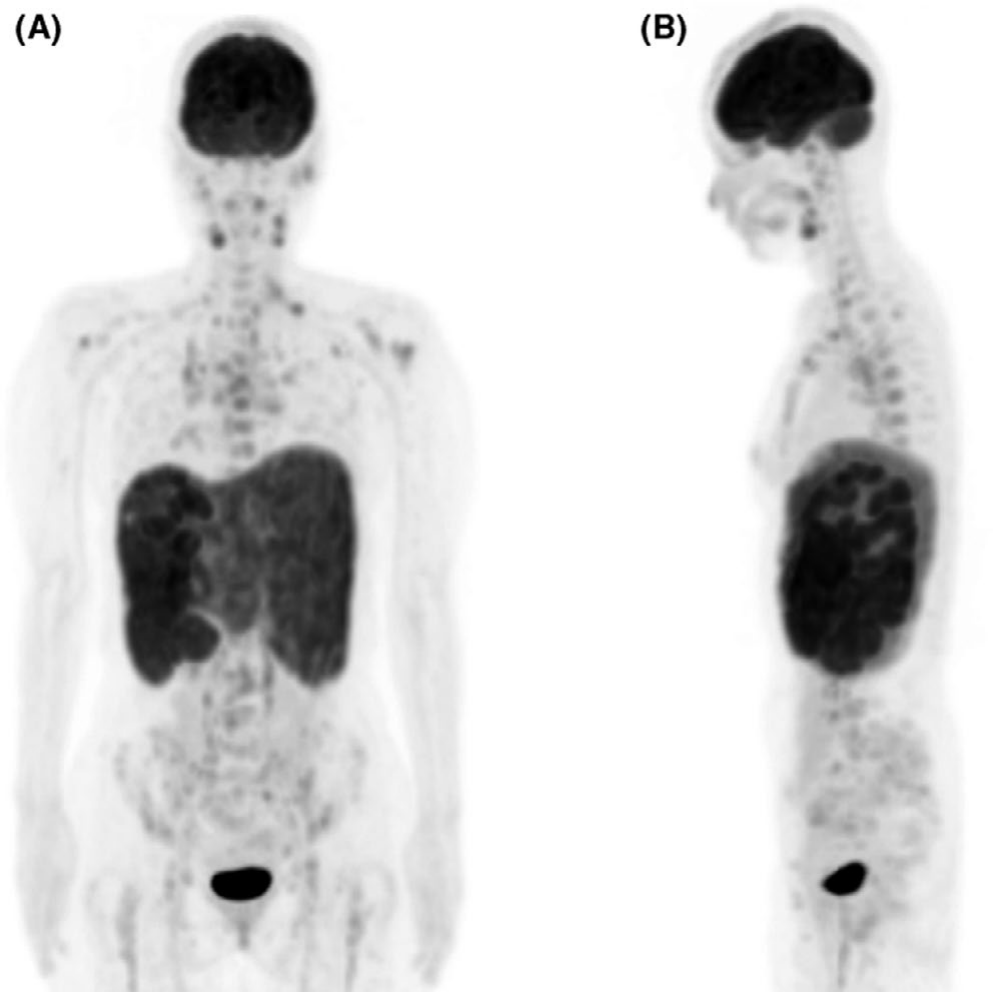
Coronal FDG PET (A) and sagittal FDG PET (B) show FDG uptake in the liver, spleen, systemic lymph nodes, humerus, scapula, the 4–7th thoracic vertebrae, pelvis, and femur

**FIGURE 2:**
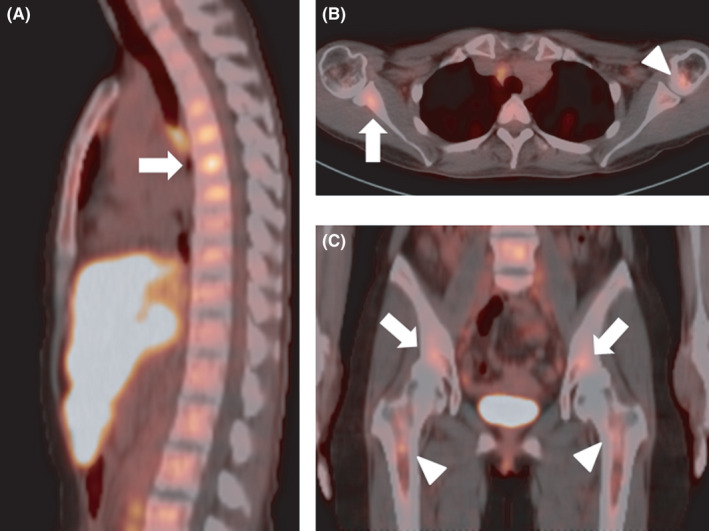
(A) Sagittal FDG PET/CT shows FDG uptake in the 4–7th thoracic vertebrae (arrow). (B) Axial FDG PET/CT shows FDG uptake in the scapula (arrow) and humerus (arrowhead). (C) Coronal FDG PET/CT shows FDG uptake in the pelvis (arrows) and femur (arrowheads)

A 48‐year‐old female patient noticed a subcutaneous nodule. She presented with remittent fever, fatigue, weight loss, and abdominal pain. Laboratory tests showed elevated levels of lysozyme, soluble interleukin‐2 receptor, and angiotensin‐converting enzyme. Computed tomography (CT) revealed pulmonary reticular opacities, hepatomegaly, and massive splenomegaly. Fluorodeoxyglucose position emission tomography (FDG PET)/CT showed FDG uptake not only in the liver, spleen, and systemic lymph nodes but also in the humerus, scapula, the 4–7th thoracic vertebrae, pelvis, and femur (Figures 1 and 2). Histological samples from the skin, liver, and bone marrow biopsies exhibited noncaseating granulomas with epithelioid cells, which were diagnosed as multiorgan sarcoidosis.

Sarcoidosis is a systemic inflammatory disease of unknown etiology characterized by the formation of noncaseating granulomas in the affected organs. Although FDG PET/CT is not included in the standard workup for sarcoidosis, its usefulness in the diagnosis of this condition and its subsequent management have been proposed.^1^ Moreover, FDG PET/CT has been reported to be especially useful in detecting bone lesions because it is difficult to detect bone sarcoidosis involvement using conventional radiography.^2^ Sarcoidosis can affect any site in the body while being asymptomatic. Therefore, FDG PET/CT should be considered for the detection of affected lesions of sarcoidosis.

## CONFLICT OF INTEREST

None.

## AUTHOR CONTRIBUTION

ST and TA contributed to the writing of this case and to the acquisition of the respective images. SH and KS reviewed and edited the manuscript.

## ETHICAL APPROVAL

None.

## CONSENT

Written informed consent was obtained from the patient for the publication of this case report and accompanying images.

## Data Availability

Data sharing not applicable to this article as no datasets were generated or analysed during the current study.
